# Multiple Metabolic Hits Converge on CD36 as Novel Mediator of Tubular Epithelial Apoptosis in Diabetic Nephropathy

**DOI:** 10.1371/journal.pmed.0020045

**Published:** 2005-02-22

**Authors:** Katalin Susztak, Emilio Ciccone, Peter McCue, Kumar Sharma, Erwin P Böttinger

**Affiliations:** 1Division of Nephrology, Department of MedicineMount Sinai School of Medicine, New York, New YorkUnited States of America; **2**Division of Nephrology, Department of MedicineAlbert Einstein College of Medicine, Bronx, New YorkUnited States of America; **3**Dorrance Hamilton Research Laboratories, Division of NephrologyDepartment of Medicine, Thomas Jefferson University, Philadelphia, PennsylvaniaUnited States of America; **4**Department of Pathology, Anatomyand Cell Biology, Thomas Jefferson University, Philadelphia, PennsylvaniaUnited States of America; Yale Medical SchoolUnited States of America

## Abstract

**Background:**

Diabetic nephropathy (DNP) is a common complication of type 1 and type 2 diabetes mellitus and the most common cause of kidney failure. While DNP manifests with albuminuria and diabetic glomerulopathy, its progression correlates best with tubular epithelial degeneration (TED) and interstitial fibrosis. However, mechanisms leading to TED in DNP remain poorly understood.

**Methods and Findings:**

We found that expression of scavenger receptor CD36 coincided with proximal tubular epithelial cell (PTEC) apoptosis and TED specifically in human DNP. High glucose stimulated cell surface expression of CD36 in PTECs. CD36 expression was necessary and sufficient to mediate PTEC apoptosis induced by glycated albumins (AGE-BSA and CML-BSA) and free fatty acid palmitate through sequential activation of src kinase, and proapoptotic p38 MAPK and caspase 3. In contrast, paucity of expression of CD36 in PTECs in diabetic mice with diabetic glomerulopathy was associated with normal tubular epithelium and the absence of tubular apoptosis. Mouse PTECs lacked CD36 and were resistant to AGE-BSA-induced apoptosis. Recombinant expression of CD36 in mouse PTECs conferred susceptibility to AGE-BSA-induced apoptosis.

**Conclusion:**

Our findings suggest a novel role for CD36 as an essential mediator of proximal tubular apoptosis in human DNP. Because CD36 expression was induced by glucose in PTECs, and because increased CD36 mediated AGE-BSA-, CML-BSA-, and palmitate-induced PTEC apoptosis, we propose a two-step metabolic hit model for TED, a hallmark of progression in DNP.

## Abstract 2

The CD36 receptor may have a key role in the degeneration of the renal tubular epithelium, the first step in diabetic nephropathy

## Introduction

Diabetic nephropathy (DNP) is a serious and common complication of type 1 and type 2 diabetes mellitus, leading to end-stage renal failure in up to 30% of individuals with diabetes. Early abnormalities of DNP affect glomeruli and include an increase in glomerular filtration rate, microalbuminuria, glomerular hypertrophy, and thickening of the glomerular basement membrane, followed by expansion of mesangial extracellular matrix and glomerulosclerosis [1,2]. As with most chronic degenerative kidney diseases, the gradual decline of renal function at later stages of DNP is invariably associated with tubular epithelial degeneration (TED), also called tubular atrophy, and interstitial fibrosis (IF), hallmarks of degeneration to end-stage renal failure [3]. Pathomechanisms that may initiate and/or mediate TED in DNP remain poorly understood. While glomerular lesions consistent with human DNP have been described in various mouse models of diabetes, TED and IF have not been described in diabetic mice [4].

Combining detailed renal phenotype analysis with gene expression profiling of hyperglycemic mouse models of type 1 (streptozotocin [STZ]) and type 2 (db/db) diabetes, we recently reported that decreased mRNA levels of CD36 in kidneys were strongly correlated with albuminuria [5]. CD36 is a transmembrane protein of the class B scavenger receptor family and is involved in multiple biological processes [6]. CD36 is widely expressed and may interact with multiple extracellular ligands, including thrombospondin-1 (TSP-1), long-chain free fatty acids (FFAs), modified (oxidized) low-density lipoprotein (ox-LDL), advanced glycation end (AGE) products, and collagens I and IV [6]. CD36 mediates phagocytosis of apoptotic cells and malaria-parasitized erythrocytes [7]. Furthermore, CD36 mediates antiangiogenic activity associated with endothelial cell apoptosis induced by TSP-1 through p38 MAP kinase (MAPK) and caspase 3 [8]. Hyperglycemia-induced synthesis of CD36 protein in macrophages has been associated with increased uptake of ox-LDL by macrophages and foam cell formation in atherosclerotic lesions in people with diabetes [6,9,10]. While diabetic cardiovascular complications are closely linked epidemiologically with albuminuria and DNP, a role for CD36 in DNP and renal pathophysiology has not to our knowledge been described to date.

Here we report a novel functional role for CD36 scavenger receptor and AGE and FFA palmitate (PA) in tubular epithelial apoptosis associated with TED and progression of DNP. Specifically, we show that glucose stimulates CD36 cell surface expression in proximal tubular epithelial cells (PTECs), and increased CD36 renders PTECs susceptible to both AGE- and PA-induced PTEC apoptosis by mediating sequential activation of src kinase, proapoptotic p38 MAPK, and caspase 3. Based on these findings, we propose a new two-step metabolic hit model for TED in the progression of DNP.

## Methods

### Animals

Kidneys were obtained from 28-wk-old C57BLKS/J-lepr^db/db^, STZ-treated C57BL/6J, or STZ-treated 129SvJ mice and from age-matched control C57BLKS/J-lepr^db/m^, C57BL/6J, and 129SvJ mice as described [5].

### Cell Culture

Human proximal tubular cell line HK-2 and murine collecting duct cell line M1 were purchased from American Type Culture Collection (Manassas, Virginia, United States) and cultured according to the vendor's instructions. Mouse proximal tubular cell line MCT was provided by Fuad Ziyadeh (University of Pennsylvania, Philadelphia, Pennsylvania, United States). Transfections were performed with Fugene 6 (Roche Diagnostics, Indianapolis, Indiana, United States) according to manufacturer's protocol. CD36-containing plasmid was a kind gift of Nada Abumhrad (SUNY at Stony Brook, New York, United States). Cells were also co-transfected with EGFP (Clontech, Franklin Lakes, New Jersey, United States) to assess transfection efficiency. Cells were serum starved in 0.2% serum containing DMEM (1 g/l glucose) for at least 24 h prior to stimulation with AGE–bovine serum albumin (BSA), glucose, or FFA.

### Quantitative Real-Time PCR

Quantitative real-time PCR analysis of mouse and human CD36, HPRT1, and beta actin was performed as described previously [5]. The following primers were used: mouse CD36 5′ TGCTGGAGCTGTTATTGGTG and 3′ CATGAGAATGCCTCCAAACA, mouse beta actin 5′ ACCGTGAAAAGATGATGACCCAG and 3′ AGCCTGGATGGCTACGTACA, mouse HPRT1 5′ TGTTGTTGGATATGCCCTTG and 3′ TTGCGCTCATCTTAGGCTTT, human CD36 5′ GCTCTGGGGCTACAAAGATG and 3′ TAGGGAGAGATATCGGGCCT, human beta actin 5′ GATGAGATTGGCATGGCTTT and 3′ CACCTTCACCGTTCCAGTTT, and human HPRT1 5′ AAAGGACCCCACGAAGTGTT and 3′ TCAAGGGCATATCCTACAACAA.

### Immunostaining and Immunoblotting

Primary antibodies specific for the following proteins were used: monoclonal mouse anti-CD36 antibody, clone FA 6–152 (IgG) (Immunotech, Fullerton, California, United States), clone SMO (IgM) (Santa Cruz Biotechnology, Santa Cruz, California, United States), rabbit polyclonal anti-CD36 (Santa Cruz Biotechnology), rabbit polyclonal anti-aquaporin1, anti-aquaporin2, anti-Na/K/2Cl (Chemicon, Temecula, California, United States), rabbit polyclonal phospho38/MAPK and mouse monoclonal p38 (Cell Signaling Technology, Beverly, Massachusetts, United States), rabbit polyclonal p-src (Y418) (Biosource, Camarillo, California, United States), and mouse monoclonal anti-tubulin (Sigma, St. Louis, Missouri, United States). Immunostaining was performed on frozen sections with FITC- and Cy3-labeled secondary antibodies (Jackson Laboratories, USA), or on paraffin-embedded sections with immunoperoxidase, as described earlier [5]. Immunoblotting was performed with 30 μg of protein isolated from cultured cells. Protein samples were resolved on a 10% SDS-PAGE and immunoblotted with primary antibody and revealed with horse radish peroxidase (HRP)-conjugated anti-mouse IgM, or anti-rabbit IgG (Jackson Laboratory, Bar Harbor, Maine, United States). Immuncomplexes were detected by enhanced chemiluminescence (Pierce, Rockford, Illinois, United States). The proximal tubular immunostaining was evaluated semi-quantitatively by two independent pathologists who were unaware of the diagnosis; distribution and intensity of staining was scored on a ten-point scale.

### Fluorescence Flow Cytometric Analysis

Cells were incubated in 0.5 mM EDTA in PBS at 37 °C for 20 min, scraped, and then washed with 1% fetal bovine serum. Cells were then exposed to monoclonal anti-CD36 IgG FA6 (5 μg/ml), or control mouse IgG1 (5 μg/ml) (Sigma), for 45 min on ice in the presence of 10% fetal bovine serum then washed with PBS. This was followed by an incubation with phycoerythrin-conjugated goat anti-murine secondary antibody (Southern Biotechnology, Birmingham, Alabama, United States) 1:50 for 45 min on ice. Cells (1 × 10^4^) were analyzed by using a SCAN flow cytometer (BD, Franklin Lakes, New Jersey, United States), with appropriate gating. Flow cytometry data were analyzed using Cellquest (BD).

### Preparation of Glycated Albumin and Carboxymethyl-Lysine Albumin

Briefly, to prepare AGE-BSA, essentially fatty-acid-free and endotoxin-free BSA (250 mg/ml) was incubated at 37 °C for 2, 5, and 10 wk with D-glucose (500 mM) in a 0.4-M phosphate buffer containing EDTA, ampicillin, Fungazone, polymixin B, and protease inhibitors. Control preparations were treated identically except that glucose was omitted. Carboxymethyl-lysine (CML)–BSA was prepared as described earlier [11]. Briefly, BSA with minimal CML content (CMLmin-BSA) was prepared by incubation of BSA (0.66 mM) with glyoxylic acid (2.15 mM) in the presence of sodium cyanoboronydrate (56 mM) in 200 mM sodium phosphate buffer (pH 7.8) at 37 °C under aseptic conditions. Finally, preparations were extensively dialyzed against phosphate buffer to remove free glucose. Preparations were then tested for the presence of LPS with a Quantitative Chromogenic LAL kit (Cambrex, East Rutherford, New Jersey, United States). The concentration of LPS was lower than 0.07 IU/mg protein in all preparations.

### Preparation of FFA

Palmitic acid (P5585), oleic acid, and FFA-free low-endotoxin BSA (A8806) were purchased from Sigma. Palmitic acid was dissolved at 12 mM in PBS containing 11% fatty-acid-free BSA, sonicated for 5 min, shaken overnight at 37 °C, and sonicated for 5 min again [12]. For control experiments, BSA in the absence of fatty acids was prepared, as described above. The effective concentration of PA was determined using a commercially available kit (Wako Chemicals, Neuss, Germany).

### Apoptosis Detection

In situ detection of DNA fragmentation was performed using the ApoTag TUNEL assay following the manufacturer's protocol (Intergen, Purchase, New York, United States) [13]. Apoptotic nuclei were detected using DAPI staining (1 μg/ml; 10 min) in cell cultures fixed with 4% paraformaldehyde, and analyzed via fluorescence microscopy to assess chromatin condensation and segregation. Caspase3 activity was detected by using the ApoAlert Caspase3 Fluorescent Detection system (BD) according to the manufacturer's protocol. Activity was normalized to total protein content. Z-DEVD-fmk, z-VAD-fmk, z-FA-fmk, and z-LEHD-fmk were purchased from BD.

### Human Kidney Biopsy Sample and Patient Characteristics

Human kidney tissues (ten controls, ten with diabetic nephropathy, and ten with focal segmental glomerulosclerosis [FSGS]) were obtained from archived kidney biopsy samples or from discarded nephrectomy specimens. All diabetic samples were from patients with biopsy-proven advanced DNP with serum creatinine ranging from 1.7 to 5.6 mg/dl (151 to 444 μM/l), heavy proteinuria (3+ by dipstick or 3–6 gr/d), and hypertension. All patients with FSGS were from patients with creatinine levels of 1.7 to 4.9 mg/dl (151 to 435 μM/l), heavy proteinuria (3+ by dipstick), and hypertension. The diagnosis of FSGS was made on Periodic acid–Schiff staining in the absence of immunodeposits on electron microscopy. The diagnosis of diabetic nephropathy was based on the presence of diabetes, proteinuria, and the characteristic light microscopy findings. Institutional Review Board approval was obtained for procurement of kidney specimens at the Thomas Jefferson University Hospital.

### Statistical Methods

Data are reported as mean and standard error of the mean (SEM) for continuous variables. All cell culture experiments were performed at least three times and summarized. Standard software packages (SPSS and Excel for Windows) were used to provide descriptive statistical plots (unpaired *t*-tests). The Bonferroni correction was used for multiple comparisons. Significance for the quantification of the CD36 staining in human biopsy samples was calculated via the Wilcoxon Rank Sum Test.

## Results

### Increased Expression of CD36 Specifically in Proximal Tubules of Human Diabetic Kidneys Is Associated with TED

**Figure 1:**
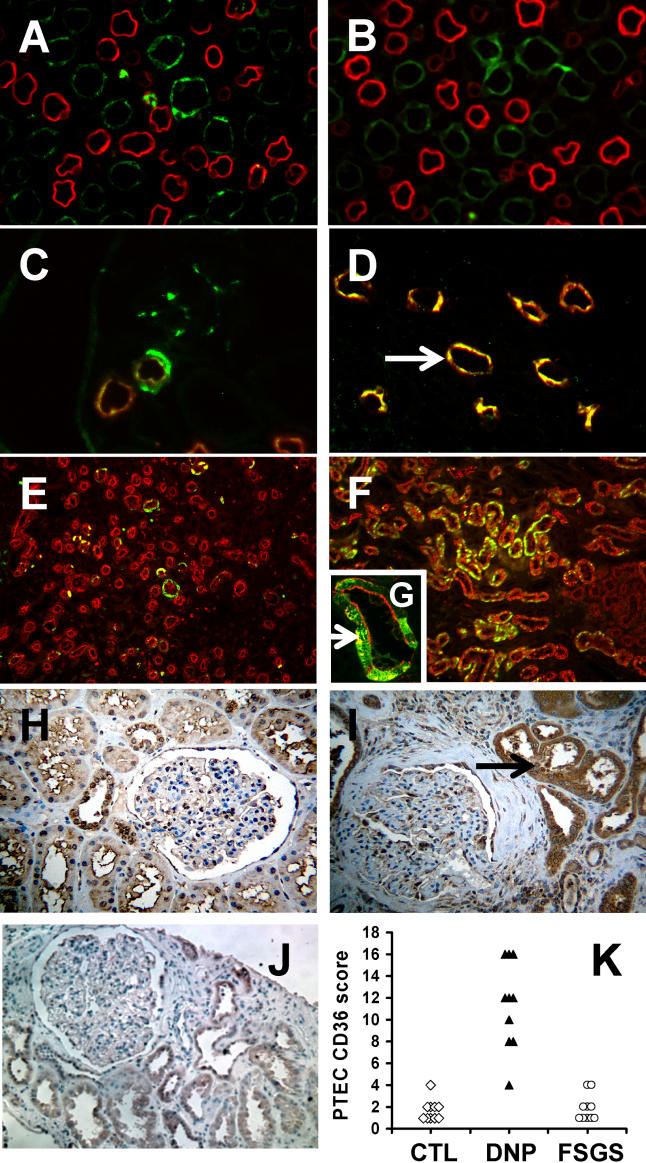
Differential Localization and Expression of CD36 Protein in Kidneys of Diabetic Mice with Glomerulopathy and of Humans with DNP (A and B) Indirect double-immunofluorescence labeling of kidney sections from non-diabetic control (A) and diabetic (B) mice with anti-CD36 (green) and proximal tubular marker anti-aquaporin1 (red). (C and D) Double labeling of non-diabetic control mice with anti-CD36 (green) and loop-of-Henle marker sodium potassium chloride cotransporter anti-NKCC (red) (C) and collecting duct marker aquaporin2 (red) (D) (arrow depicts colocalization of anti-CD36 and anti-aquaporin2 staining). (E and F) Double labeling of human kidney sections from control individuals (E) and individuals with diabetes with DNP (F) using anti-CD36 (green) and anti-aquaporin1 (red). (G) Higher-magnification image of (F) with arrows depicting colocalization of anti-CD36 and anti-aquaporin1. (Note that anti-CD36 labeling is heterogeneous: staining is isolated proximal tubular cells.) (H–J) Representative images of anti-CD36 immunoperoxidase staining of sections of normal human kidney (H), human kidney with DNP (I), and human kidney with FSGS (J). Arrow in (I) depicts proximal tubular epithelial staining. (K) CD36 PTEC expression score derived from blinded, semi-quantitative analysis of distribution and intensity of proximal tubular CD36 staining of human biopsy samples from ten normal control, ten DNP, and ten FSGS kidneys and the result shown on a dot plot. Significance was calculated by Wilcoxon Rank Sum Test, and PTEC scores for DNP kidneys were significantly different from those of FSGS kidneys and normal human kidneys.

Using microarray-based gene expression profiling on whole kidney RNA together with supervised clustering methods, we previously identified and validated gene expression patterns for molecular classification of diabetic mice with albuminuria and mesangial expansion [5]. Reduced renal mRNA levels of the class B scavenger receptor CD36 were characteristic for diabetic mice with albuminuria [5]. Here we examined patterns of CD36 protein expression in kidneys of non-diabetic and diabetic mice and humans. CD36 protein was detectable in the thick ascending limb of loop of Henle and in the collecting duct, and absent in proximal tubules in both control and diabetic mouse kidneys (Figure 1A–1D). In contrast, CD36 was detectable only rarely in individual proximal tubular cells in sections from non-diabetic human kidneys (controls) (Figure 1E and 1H), but was markedly increased specifically in PTECs in human diabetic kidneys (Figure 1F and 1I). In addition, we did not observe increased proximal tubular CD36 expression in kidney biopsy samples from patients with FSGS (Figure 1J), that were matched with DNP samples for the severity of proteinuria (all in the nephrotic range) and renal insufficiency (all with elevated serum creatinine; 1.7–5.0 mg/dl). Semi-quantitative analysis of the distribution and intensity of CD36-positive PTECs (CD36 PTEC score), which was performed by two independent pathologists in a blinded manner, demonstrated that mean CD36 PTEC scores were not different between FSGS kidneys and normal human kidneys, but were significantly increased in DNP kidneys (Figure 1K).

**Figure 2:**
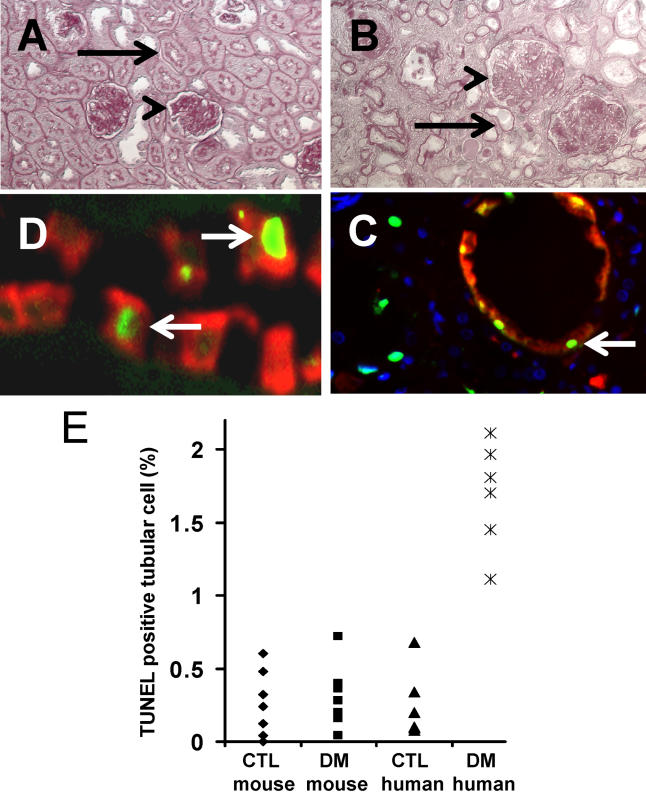
TED and IF Coincide with Proximal Tubular Apoptosis and CD36 Expression in Human DNP (A and B) Periodic Acid–Schiff staining of diabetic mouse kidney (28-wk-old C57BLKS/J-lepr^db/db^) (A) and human DNP kidney (B). Arrowheads denote glomeruli with advanced mesangial expansion and glomerulosclerosis; arrows depict normal proximal tubule in diabetic mouse (A) and TED in human with DNP (B). (C) TUNEL assay (green) and anti-CD36 (red) double labeling of human DNP. Arrows indicate apoptotic, CD36-positive tubular epithelial cells. (D) TUNEL assay (green) and anti-aquaporin1 (red) double labeling of human DNP. Arrows depict TUNEL-positive and aquaporin1-positive PTECs. (E) Dot plot indicates the number of TUNEL-positive tubular cells per 100 total tubular cells in kidneys of control (CTL) and diabetic (DM) mice and humans, as indicated.

Periodic acid–Schiff–stained sections of kidneys from mice exposed to type 2 diabetes (db/db mice) for 20 wk (Figure 2A), or type 1 diabetes (STZ-treated C57BL/6J mice) for 20 wk (data not shown) demonstrated moderate to advanced mesangial expansion and glomerulosclerosis (Figure 2A). Tubular abnormalities were not detectable in either model (Figure 2A). In contrast, TED and IF were associated with moderate to advanced mesangial expansion and glomerulosclerosis on kidney sections of human DNP (Figure 2B). These findings indicate that in humans with DNP, diabetes-induced upregulation of CD36 expression in proximal tubules was associated with moderate to advanced stages of TED and IF. In contrast, in diabetic mice with albuminuria, mesangial expansion, and glomerulosclerosis, absence of CD36 expression was associated with normal appearance of the tubular epithelium and interstitial space. These findings suggest an association between diabetes-induced proximal tubular CD36 expression and TED.

### Coincidence of Increased CD36 Expression and Increased Tubular Epithelial Cell Apoptosis in Human DNP

CD36 has been shown to mediate apoptosis signaling induced by TSP-1 in endothelial cells [8] and by ox-LDL in macrophages [14]. We examined whether the strong upregulation of CD36 protein in PTECs, observed specifically in human DNP, was associated with tubular epithelial cell apoptosis in vivo. TUNEL-positive tubular epithelial cells also stained positive for CD36 protein (Figure 2D) and aquaporin1 (Figure 2C), indicating that apoptosis and CD36 expression coincided in PTECs in human DNP. In contrast, CD36 was not detectable in TUNEL-positive PTECs in non-diabetic FSGS kidneys and in normal human kidney (data not shown). Statistical analysis showed that the rate of TUNEL-positive tubular cells was significantly increased in kidneys of human DNP compared with normal control human kidney (Figure 2E). In addition, tubular epithelial apoptosis was increased, but highly variable, in FSGS kidneys (data not shown). In contrast, tubular epithelial apoptosis rates were comparable between non-diabetic control and all diabetic mouse kidneys (Figure 2E). The diabetic mouse group included 24-wk-old STZ-treated diabetic C57BL/6J or 129SvJ mice (0.23 ± 0.1 TUNEL-positive cells per 100 tubular cells) and 24-wk-old lepr^db/db^ mice (0.2 ± 0.1 TUNEL-positive cells per 100 tubular cells). Together, these findings indicate that CD36 expression in PTECs is associated with apoptotic events of proximal tubular cells and TED specifically in human DNP, but not in FSGS with matched functional and clinical abnormalities. These in vivo findings demonstrate a strong association of diabetes-induced CD36 expression and apoptosis in PTECs in human DNP, suggesting that CD36 may play a critical role in TED by mediating PTEC apoptosis in progressive human DNP.

### High Ambient Glucose Induces CD36 Expression in Human PTECs

**Figure 3:**
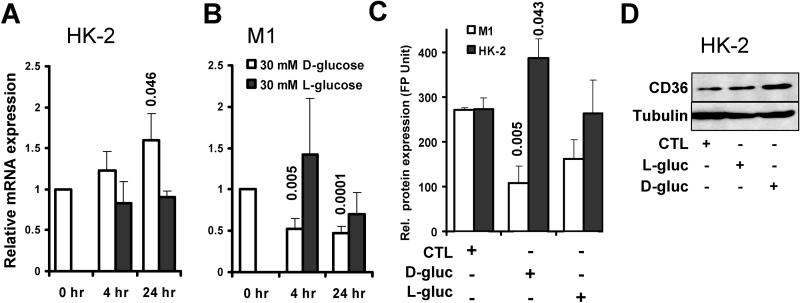
CD36 mRNA and Protein Synthesis Is Stimulated in Human, but Not in Murine, PTECs, and Is Suppressed in Murine Collecting Duct Cells by High Ambient Glucose (A) Relative CD36 mRNA abundance determined by quantitative real-time PCR in human PTEC line HK-2 treated with 30 mM D-glucose (open bars) or control L-glucose (black bars) for 4 and 24 h following maintenance of cells in 5 mM D-glucose medium. Bars represent mean ± SEM of three to five repeat experiments. Numbers on top of bars indicate significant *p*-values of experimental groups relative to 0 h. (B) Bar graphs show experiment as described under (A), using mouse collecting duct cell line M1 instead of human HK-2 PTECs. The relative expression of CD36 was normalized to internal control housekeeping genes *HPRT* and *beta actin,* and to baseline controls (untreated cells). (C) Relative cell surface expression of CD36 protein determined by FACS in M1 cells (open bars) and HK-2 cells (black bars) maintained in 5 mM D-glucose medium (CTL), or in medium containing 30 mM D-glucose (D-gluc) or L-glucose (L-gluc) for 72 h. (Original FACS histograms are provided in Figure S1.) Bars represent mean ± SEM of three to five repeat experiments. Numbers indicate significant *p*-values of experimental groups relative to control. (D) Immunoblot showing CD36 protein levels in human HK-2 PTECs maintained in control 5 mM D-glucose (CTL), or after stimulation for 72 h with 30 mM L-glucose (L-gluc) or D-glucose (D-gluc), as indicated. Tubulin is shown for loading control. All data represent at least four independent repeat experiments.

High ambient glucose has been shown to induce CD36 protein synthesis in macrophages [9]. Because CD36 protein was markedly increased in proximal tubules in human DNP, we examined the effects of high ambient glucose on CD36 mRNA and protein expression in the human PTEC line HK-2 (Figure 3). Exposure of cells to 30 mM D-glucose for 24 h, but not to control L-glucose, significantly increased levels of CD36 mRNA (Figure 3A), CD36 cell surface protein (Figure 3C), and CD36 protein expression in cell lysates (Figure 3D). In contrast, CD36 mRNA and protein were not detectable in the murine PTEC line MCT at either normal or high ambient glucose concentrations (data not shown). Interestingly, glucose stimulation decreased CD36 mRNA levels (Figure 3B) and CD36 cell surface protein (Figure 3C) in the murine collecting duct cell line M1, consistent with our previously reported findings in diabetic mouse kidney [5]. Exposure of human HK-2 and murine M1 cell lines to defined preparations of FFA PA or AGE-BSA had no effect on CD36 mRNA and protein expression levels (data not shown). These findings demonstrate that high ambient glucose causes upregulation of CD36 mRNA and protein specifically in human, but not in mouse, PTECs. Together with our in vivo observations, these results suggest that hyperglycemia may induce upregulation of CD36 mRNA and protein selectively in proximal tubules in kidneys of human DNP, but not diabetic mice with albuminuria.

### AGE-BSA, CML-BSA, and FFA PA Induce Apoptosis in Human PTECs via CD36

**Figure 4:**
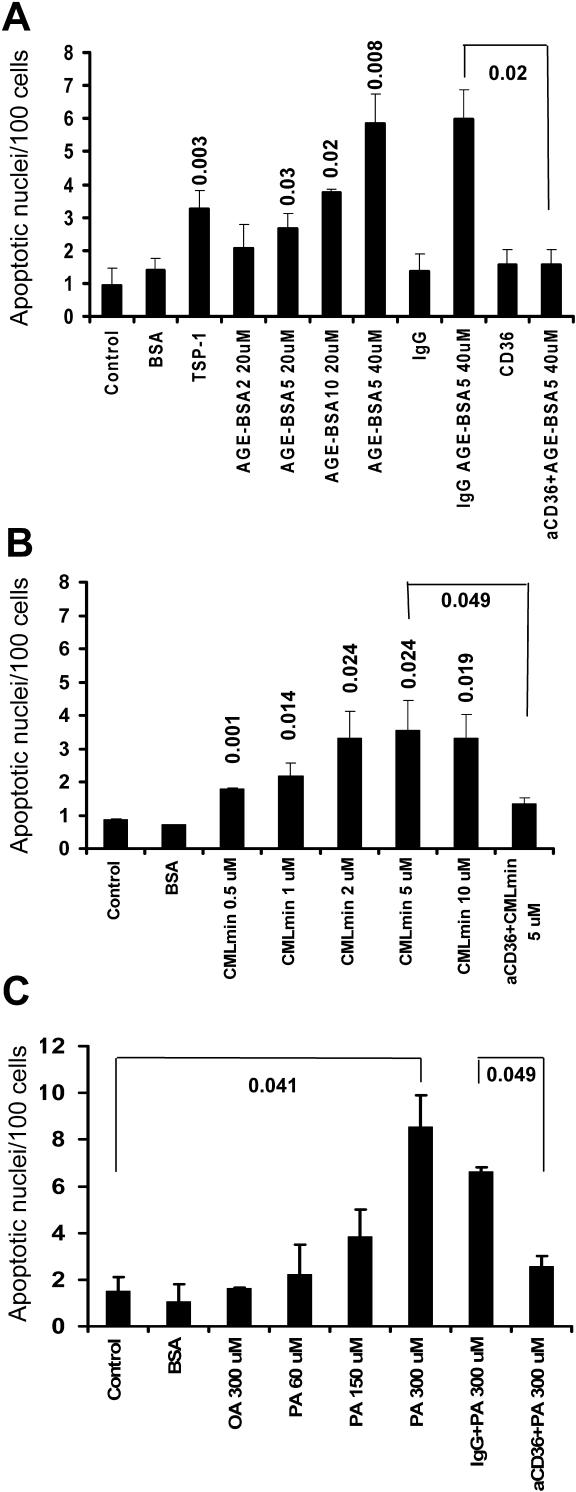
AGE-BSA, CML-BSA, and FFA PA Induce Apoptosis in Human PTECs through CD36 Signaling Bar graphs show mean ± SEM of apoptotic nuclei, visualized by DAPI staining and normalized to 100 total cells, in human HK-2 PTECs. Data are derived from three independent repeat experiments. Numbers on top of bars indicate significant *p*-values of experimental groups relative to control, or as indicated by bracket. (A) Cells were treated for 48 h with control BSA (40 μM), TSP-1 (1 μg/ml), and AGE-BSA modified for 2, 5, or 10 weeks (AGE-BSA2, AGE-BSA5, and AGE-BSA10, respectively) in the absence or presence of control IgG (10 μg/ml) or anti-CD36 neutralizing antibody (10 μg/ml), as indicated. (B) Cells were treated with control BSA (40 μM), or CMLmin-BSA at 0.5, 1, 2, 5, and 10 μM, in the absence or presence of anti-CD36 neutralizing antibody, as indicated. (C) Cells were treated with monounsaturated FFA oleic acid (OA) or PA at increasing concentrations, in the absence or presence of control IgG (10 μg/ml) or anti-CD36 neutralizing antibody (10 μg/ml), as indicated.

AGE albumin [15] and FFAs [16] have been implicated in the pathogenesis of diabetic complications, including tubular degeneration [17] and tubular epithelial-to-mesenchymal transition [18]. In addition, AGE albumin and FFA are known to interact with CD36 [19,20]. However, it is not known whether AGE and/or FFA can activate CD36 signaling and apoptosis in tubular epithelial cells. Treatment with AGE-BSA for 2, 5, or 10 wk or with CML-BSA induced a significant increase in the number of apoptotic nuclei in CD36-positive HK-2 cells compared with control BSA-treated or untreated HK-2 cells (Figure 4A). In contrast, AGE-BSA and CML-BSA had no effect on the rate of apoptotic nuclei in CD36-negative murine MCT PTECs (data not shown). Because AGE-BSA glycated for 5 wk (AGE-BSA5) induced robust apoptosis at concentrations between 20 and 40 μM (Figure 4A), we chose this preparation and concentration for further analysis in all subsequent experiments. AGE-BSA5-induced apoptosis was blocked when cells were preincubated with neutralizing anti-CD36 antibody, while preincubation with control IgG antibody had no effect (Figure 4A). These results were confirmed by DNA laddering assay (data not shown).

Among the most abundant glucose-modified proteins detectable in the plasma of diabetic individuals are CML proteins [21], which are typically present at 1.6 to 2.3 μM concentrations in the plasma and urine of diabetic individuals [22,23]. To use physiologically relevant CML proteins in our in vitro experiments, we prepared CMLmin-BSA, characterized by glycation of approximately 30% of lysine residues [21]. When applied to HK-2 PTECs at concentrations ranging from 0.5 to 10 μM, CMLmin-BSA increased apoptosis rates significantly (Figure 4B). The proapoptotic effect of CMLmin-BSA was blocked by CD36 neutralizing antibody, but not by control IgG (Figure 4B).

CD36 has been shown to transport fatty acids in adipocytes [24] and in muscle cells [25]. Concentrations of FFAs may be substantially elevated, to levels of up to 700 μM, in individuals with type 2 diabetes or obesity [26]. Thus, we examined the effects of saturated FFA PA and monounsaturated FFA oleate on apoptosis of HK-2 PTECs in the absence or presence of anti-CD36 neutralizing antibody. PA significantly increased rates of apoptotic nuclei in a concentration-dependent manner in HK-2 PTECs (Figure 4C). Anti-CD36 neutralizing antibody, but not control IgG, blocked PA-induced apoptosis (Figure 4C). In contrast, oleate did not induce apoptosis, even at concentrations as high as 300 μM (Figure 4C), neither did it prevent PA-induced apoptosis (data not shown). Of note, these experiments were performed using a total fatty acid:BSA ratio of 6.6:1, in order to closely model pathophysiologic states in which unbound FFA concentration is high [27]. Taken together, our findings demonstrate that pathophysiologically relevant species of AGE-BSA and CML-BSA, as well as saturated FFA PA, induce apoptosis in human PTECs at concentrations previously observed in plasma and/or urine in humans with diabetes.

### AGE-BSA and PA Sequentially Activate src kinase, Proapoptotic p38 MAPK, and Caspase 3 through CD36 Receptor

**Figure 5:**
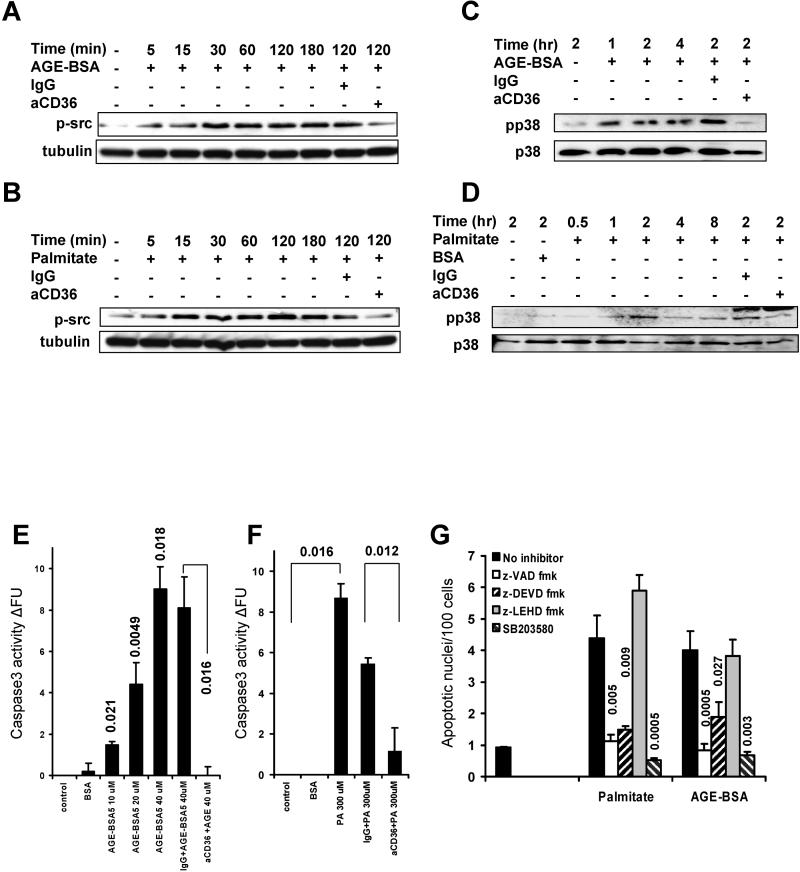
Activation of Intracellular Pathways following AGE-BSA and PA Treatment of Human HK-2 PTECs (A and C) Immunoblots show levels of (A) phosphorylated (Y418) src kinase and tubulin or (C) phosphorylated p38 MAPK (pp38) and total p38 MAPK (p38) in HK-2 cells treated with AGE-BSA5 (40 μM) in the absence or presence of control IgG or anti-CD36 neutralizing antibody (10 μg/ml) for different time periods, as indicated. (B and D) As shown in (A) and (C), except HK-2 cells were treated with PA (150 μM) instead of AGE-BSA5. (E and F) Bar graphs demonstrate mean ± SEM of caspase 3 activity in three independent repeat experiments. Caspase 3 activity was measured by quantitative ELISA in HK-2 cells after 18 h of stimulation with AGE-BSA5 and PA, as per manufacturer's protocol. Numbers on top of bars indicate significant *p*-values of experimental groups relative to control, or as indicated by brackets. (G) Bar graphs demonstrate number of apoptotic nuclei of HK-2 cells, normalized to 100 total cells, treated with AGE-BSA5 (40 μM) or PA (150 μM) in the absence (black bars) or presence of pan-caspase inhibitor (z-VAD-fmk [100 μM]; open bars), caspase 3 inhibitor (z-DEVD-fmk [20 μM]; first striped bars), caspase 9 inhibitor (z-LEHD-fmk (20 μM); gray bars), or chemical inhibitors of p38 MAPK (SB203580 [10 μM]; second striped bars). Mean ± SEM of three independent repeat experiments is presented. Numbers on top of bars indicate the significant *p*-values for comparison relative to control (no inhibitor).

CD36 has previously been shown to trigger the activation of p59fyn, p38 MAPK, and caspase 3 (GeneID: 836) in response to thrombospondin in endothelial cells [8]. Therefore we examined phospho-src, phospho-p38 levels and caspase 3 activation in HK-2 PTECs treated with AGE-BSA and PA in the absence or presence of anti-CD36 neutralizing antibody. Both AGE-BSA5 and PA increased phospho-src levels rapidly (after as little as 5 min), and over a prolonged time interval (up to 3 h) (Figure 5A and 5B). Phosphorylation of src kinase was blocked by anti-CD36 neutralizing antibody (Figure 5A and 5B). This observation is consistent with previous findings demonstrating direct interaction between CD36 and p59fyn [8]; however, the involvement of other src kinases cannot be excluded. We also observed increased levels of phosphorylation of p38 MAPK beginning 1 to 2 h after treatment, and p38 activation was also completely blocked by anti-CD36 neutralizing antibody (Figure 5C and 5D). These findings indicate that CD36 activates proapoptotic p38 MAPK possibly via src kinase activation in human PTECs when stimulated with AGE-BSA5 and PA. Chemical inhibition of p38 MAPK prevented the increase in the rate of apoptotic nuclei induced by both AGE-BSA5 and PA in HK-2 PTECs (Figure 5G), indicating that p38 MAPK function is required for apoptosis induced by AGE-BSA and PA through CD36 receptor. AGE-BSA and PA significantly increased activity of effector caspase 3 in human PTECs (Figure 5E and 5F). Caspase 3 activation was blocked by anti-CD36 neutralizing antibody, but not by control IgG (Figure 5E and 5F). Pan-caspase inhibitor z-VAD-fmk and the specific caspase 3 inhibitor z-DEVD-fmk prevented apoptosis induced by PA and AGE-BSA, while the specific caspase 9 inhibitor z-LEHD-fmk had no significant inhibitor effect (Figure 5G). Together these findings indicate that CD36 receptor mediates sequential phosphorylation of src kinases and p38 MAPK, leading to activation of caspase 3 and apoptosis in human PTECs exposed to AGE-BSA and PA ligands. Interestingly, we did not observe phosphorylation of Smad2 and p42/44 ERK MAPK under these conditions, as previously reported for AGE binding to the RAGE receptor [28].

### CD36 Is Sufficient to Mediate Apoptosis Induced by AGE-BSA and FFA

**Figure 6:**
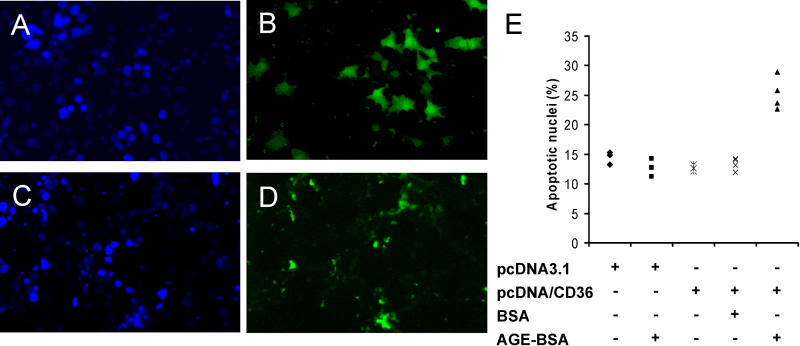
Expression of CD36 Transgene Confers Susceptibility to AGE-BSA-Induced Apoptosis (A–D) Representative images show DAPI (A and C) and FITC (B and D) labeling of CD36-negative MCT cells treated with 40 μM AGE-BSA5 for 24 h after co-transfection with green fluorescent protein plasmid pEGFP and pcDNA3.1 empty control vector (A and B), or pEGFP and CD36 expression plasmid pcDNA3.1/CD36 (C and D). (E) The dot plot shows results of four independent experiments where apoptotic nuclei per 100 total cells were quantitated in transfected cell cultures with or without treatments as indicated.

In contrast with CD36-positive human HK-2 PTECs, we found that treatment of CD36-negative mouse MCT PTECs with AGE-BSA had no effect on rates of apoptotic nuclei (data not shown). To test whether CD36 was sufficient to mediate AGE-BSA-induced apoptosis, we transfected CD36-negative mouse MCT PTECs with a plasmid expressing human CD36 or empty control vector, followed by treatment with control BSA or AGE-BSA5. AGE-BSA5 treatment had no significant effect on rates of apoptotic nuclei in MCT PTECs transfected with control vector (Figure 6). In contrast, AGE-BSA significantly increased apoptotic nuclei compared with unglycated BSA in MCT PTECs transiently transfected with CD36 expression vector (Figure 6). Nonglycated control albumin did not cause apoptosis. Thus, transgenic de novo expression of human CD36 in CD36-negative mouse PTECs was sufficient to mediate apoptosis induced by AGE-BSA.

## Discussion

Advanced diabetic nephropathies in humans with type 1 or type 2 diabetes are uniformly characterized by TED, or tubular atrophy, and IF leading to renal failure [29,30]. Although TED and IF are the strongest predictors for progression of DNP [31], mechanisms that underlie TED in DNP remain poorly understood. Based on our in vitro and in vivo findings we propose a two-step metabolic hit model for TED in DNP. High ambient glucose, but not AGE or FFA, cause stimulation of CD36 expression in PTECs specifically in diabetic kidneys. Increased CD36 expression mediates sequential activation of src kinase, proapoptotic p38 MAPK, and caspase 3 in PTECs in the presence of AGE and FFA PA, resulting in PTEC apoptosis. Proximal tubular epithelial apoptosis may be an initial mechanism for TED in DNP.

Our conclusions are supported by several key observations. First, we identify a new functional role for CD36 as an essential mediator of proximal tubular epithelial apoptosis, inducible by AGE-BSA, CMLmin-BSA, and FFA PA. Previous reports demonstrated a role for CD36 in mediating apoptosis induced by TSP-1 in endothelial cells and ox-LDL in macrophages [8,14]. In the present study, we show for the first time, to our knowledge, that CD36 mediates apoptosis in differentiated epithelial cells that are exposed to AGE-BSA-, CMLmin-BSA-, and FFA-induced metabolic injury characteristic of the diabetic milieu. Interestingly, AGE albumins and CML are present in the urine of individuals with diabetes with albuminuria due to DNP and have been shown to bind proximal tubular epithelium [22,32]. While the presence or absence of FFAs in the urine of diabetics with DNP has not been determined to date, FFAs may cause tubular apoptosis [33]. It remains to be determined whether FFA interacts with CD36 to activate CD36 receptor signaling, or whether CD36 mediates FFA uptake to activate src kinase and p38 MAPK signaling. Irrespective of the upstream mechanism of FFA and CD36 interaction, our results demonstrate very rapid activation of a well-characterized intracellular kinase cascade of proapoptotic signaling. Our finding that AGE-BSA and PA induce apoptosis through a CD36-mediated and p38- and caspase-dependent mechanism in tubular epithelial cells, similar to TSP-1 and ox-LDL in endothelial cells and macrophages, respectively, suggests that multiple, context-dependent extracellular stimuli of apoptosis may converge on CD36 scavenger receptor to activate src kinase and proapoptotic p38 MAPK pathway. In the context of the diabetic milieu and diabetic complications, our findings provide new molecular insights into diabetes-induced AGE- and FFA-dependent injury of renal epithelial cells.

Almost all TUNEL-positive apoptotic tubular epithelial cells showed increased expression of CD36, suggesting a strong correlation between upregulation of CD36 expression and increased apoptosis in PTECs specifically in human diabetic kidney in vivo. Importantly, biopsy samples from cases of FSGS that were matched for degree of proteinuria, renal function, and hypertension were characterized by TED, IF, and increased tubular epithelial apoptosis; however, proximal tubular CD36 expression was similar to that in normal human control kidney. Therefore, CD36 expression in PTECs is specifically associated with the diabetic condition and appears to be independent of degree of proteinuria and renal failure. Indeed, increased CD36 expression in PTECs in human DNP in vivo may be caused by hyperglycemia, as we show that high glucose concentration stimulates CD36 expression in vitro. It is intriguing that CD36 expression was not detected in PTECs in diabetic mice with longstanding hyperglycemia in vivo, although underlying mechanisms for the species-dependent differential regulation of CD36 in PTECs in vivo and in vitro between mouse and human remain unclear at this time. Comparisons of human *CD36* and mouse *Cd36* genes indicate a high degree of sequence and structural similarity in both coding and regulatory regions, suggesting that the mechanism or mechanisms that underlie our findings are likely determined by sequence-independent, epigenetically distinct response patterns to the diabetic milieu that differ between these species. It is also possible that dietary or metabolic factors account for the differences in CD36 expression, as mice were maintained on standard mouse chow characterized by significantly lower fat and cholesterol contents than typical western diets consumed by humans. However, dietary or other unknown environmental factors cannot explain the differential CD36 regulation by glucose in human and mouse PTECs. Thus, we are exploring whether biochemical or functional differences between mouse and human PTECs in glucose metabolism or glucose-induced signaling can be identified. However, current lack of understanding of the observed differential regulation between human and mouse does not diminish the translational research significance of our findings, with their clear therapeutic implications. Thus, the present study identifies a new CD36-dependent molecular signaling pathway that mediates tubular epithelial apoptosis, and may underlie TED and IF, hallmarks of disease progression, specifically in human diabetic nephropathy.

Third, to our knowledge, our report provides the first controlled study demonstrating increased apoptosis specifically in PTECs in DNP with TED and IF. These findings are consistent with a recent uncontrolled case series of five patients with DNP [34], and with previous reports demonstrating tubular apoptosis in kidneys of STZ-treated diabetic rats [35,36]. Interestingly, our study shows that tubular epithelial apoptosis was associated with TED and IF in human DNP, while normal appearance of tubular epithelium and interstitium was associated with baseline apoptosis rates in diabetic mouse models. Together, published observations from experimental diabetes models in mouse and rat, and human DNP, and our own findings in diabetic mouse models and human DNP, suggest a striking association of TED and tubular epithelial apoptosis. However, whether tubular epithelial apoptosis causes TED in DNP will require further investigation. Interestingly, acute and chronic chemical inhibition of caspase activity in a nephrotoxic serum nephritis model of chronic progressive glomerulonephritis with TED and IF reduced tubular apoptosis and TED [37]. Decreased tubular apoptosis and TED were associated with significantly reduced IF and decreased collagen synthesis in this model. This finding suggests that tubular epithelial apoptosis may trigger TED and IF in this model of chronic glomerulonephritis in rat, and supports our conclusions that diabetes-induced tubular epithelial apoptosis may underlie TED associated with IF in human DNP.

In conclusion, we report a new functional role for CD36 scavenger receptor in tubular epithelial apoptosis associated with tubular degeneration and progression of DNP. Specifically, we show for the first time that both AGE and FFA PA induce PTEC apoptosis through CD36-mediated activation of src kinase, p38 MAPK, and caspase 3. Because high glucose stimulates CD36 expression in human PTECs and because CD36 expression is increased in apoptotic tubular epithelial cells in human DNP, we propose a two-step metabolic hit model relevant for TED, a hallmark of progression of human DNP.

## Supporting Information

Figure S1 Glucose Regulates CD36 Expression in Tubular Cells Flow cytometric analysis of (A) human (HK-2) and (B) murine (M1) tubular epithelial cells incubated with control IgG (green curve) or with anti-CD36 antibody (FA6) (black curve) in medium containing 5 mM glucose (empty curve) or in medium containing 30 mM glucose (red curve) for 3 d.
(45 KB PPT).

### Accession Numbers

The LocusLink (http://www.ncbi.nlm.nih.gov/LocusLink/) accession numbers for the gene products discussed in this paper are caspase 3 (GeneID: 836), CD36 (GeneID: 948), MAPK (GeneID: 1432), p42/44 ERK MAPK (GeneID: 50689), p59fyn (GeneID: 2534), and Smad2 (GeneID: 4087).

**Patient Summary**

**Background**

The kidneys are often affected in people with diabetes. Around one in three people with type 1 (juvenile, or insulin-dependent) and one in ten people with type 2 (late onset, or non-insulin-dependent) diabetes will develop kidney disease (called diabetic nephropathy). Diabetic nephropathy is one of the leading complications of diabetes and is the leading cause of kidney failure worldwide. Some risk factors make it more likely that certain people with diabetes will develop kidney disease—for example, kidney disease occurs more often in patients from South Asian or African backgrounds, in men, in patients with poor control of their blood sugar levels, and in those with high blood pressure or who smoke. However, the details of how, exactly, diabetes damages the kidneys are not clear.

**What Did the Investigators Do?**

They studied samples taken from the kidneys of humans and mice with and without diabetes and looked at the effects of high glucose concentrations on the cells in the kidneys. They found that in one part of the human kidneys high glucose caused a change in the cell surface causing an increase in a protein called CD36. This change occurred in the samples from people with diabetes, but did not occur in the samples from mice with diabetes. The investigators also found that some substances that are often found in the blood of people with diabetes could join to CD36; in doing so, these substances triggered the death of these cells, which is one of the first steps that occurs in diabetic nephropathy.

**What Do These Findings Mean?**

This particular protein (CD36) could have a central role in triggering diabetic nephropathy. Although there are no immediate clinical implications of this research for the treatment of people with kidney problems, this research helps in understanding how high glucose damages the kidney. In particular, it highlights how important it is to keep blood glucose levels as normal as possible.

**Where Can I Get More Information?**

Medline Plus's article on diabetic nephropathy: http://www.nlm.nih.gov/medlineplus/ency/article/000494.htm

Diabetes UK's online information centre: http://www.diabetes.org.uk/infocentre/index.html

National Diabetes Information Clearinghouse: http://diabetes.niddk.nih.gov/

National Institute of Diabetes and Digestive and Kidney Diseases (NIDDK) Animal Models of Diabetic Complications Consortium (AMDCC): http://www.amdcc.org/

## Abbreviations

AGE: advanced glycation end
AGE-BSA5: advanced glycation end–bovine serum albumin glycated for 5 wk
BSA: bovine serum albumin
CML: carboxymethyl-lysine
CLMmin: bovine serum albumin with minimal carboxymethyl-lysine content
DNP: diabetic nephropathy
FFA: long-chain free fatty acid
FSGS: focal segmental glomerulosclerosis
IF: interstitial fibrosis
MAPK: MAP kinase
ox-LDL: modified (oxidized) low-density lipoprotein
PA: palmitate
PTEC: proximal tubular epithelial cell
SEM: standard error of the mean
STZ: streptozotocin
TED: tubular epithelial degeneration
TSP-1: thrombospondin-1
