# Supplementary material for: Multiple Metabolic Hits Converge on CD36 as Novel Mediator of Tubular Epithelial Apoptosis in Diabetic Nephropathy
Source: PLoS Med. 2005 Feb 22;2(2):e45. doi: 10.1371/journal.pmed.0020045 (PMC549593; doi:10.1371/journal.pmed.0020045)
Supplement: Figure S1 — Flow cytometric analysis of (A) human (HK-2) and (B) murine (M1) tubular epithelial cells incubated with control IgG (green curve) or with anti-CD36 antibody (FA6) (black curve) in medium containing 5 mM glucose (empty curve) or in medium containing 30 mM glucose (red curve) for 3 d. (45 KB PPT). [file pmed.0020045.sg001.ppt]

## Slide 1
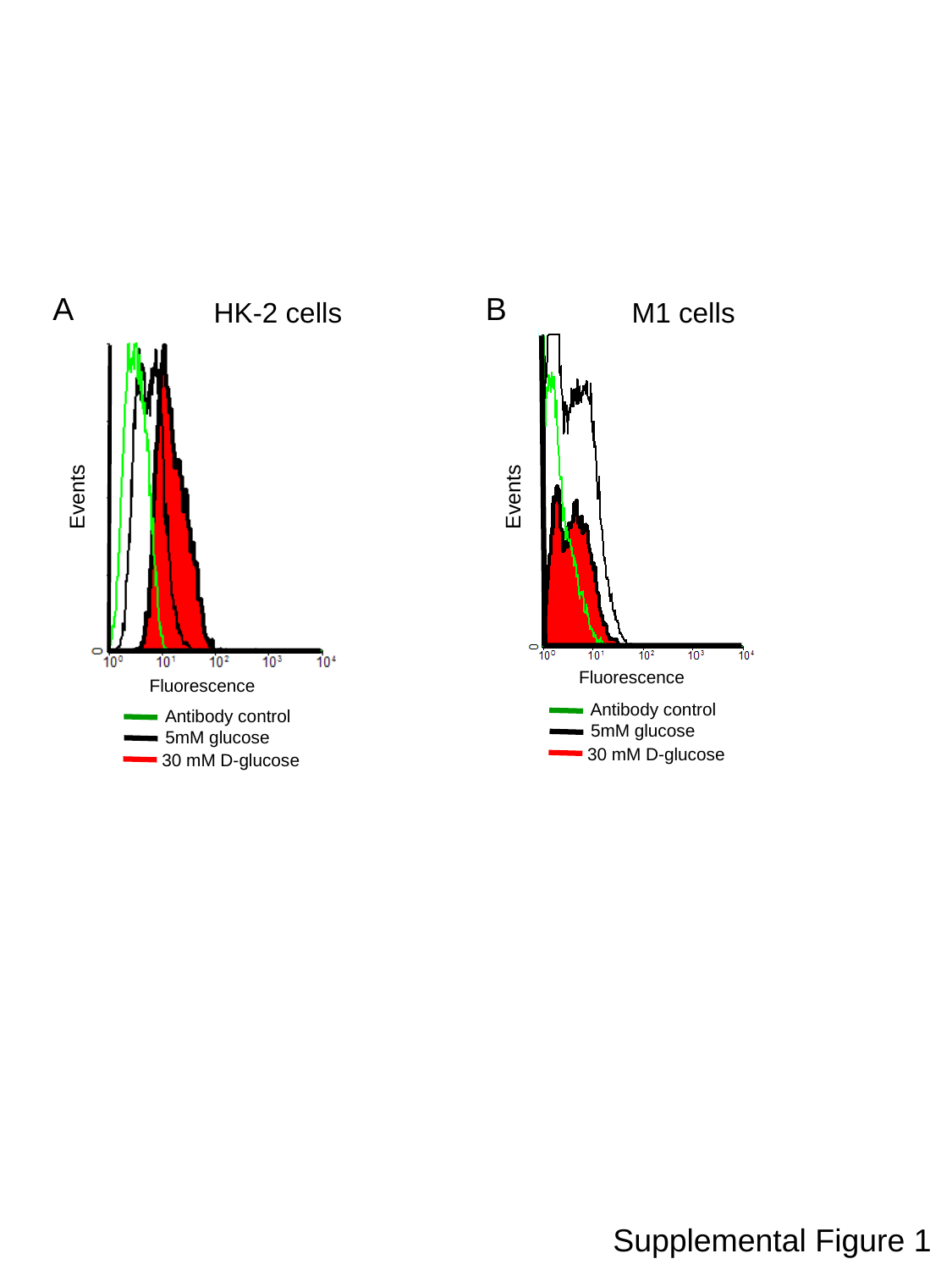

A
B
HK-2 cells
M1 cells
Events
Events
Fluorescence
Fluorescence
Antibody control
Antibody control
5mM glucose
5mM glucose
30 mM D-glucose
30 mM D-glucose
Supplemental Figure 1
